# Discriminative imaging of maternal and fetal blood flow within the placenta using ultrafast ultrasound

**DOI:** 10.1038/srep13394

**Published:** 2015-09-16

**Authors:** Bruno-Felix Osmanski, Edouard Lecarpentier, Gabriel Montaldo, Vassilis Tsatsaris, Pascale Chavatte-Palmer, Mickael Tanter

**Affiliations:** 1 Institut Langevin, ESPCI ParisTech, PSL University, CNRS UMR7587, INSERM U979, Université Paris VII, France; 2 UMR-S 1139 INSERM, Paris Descartes University, Sorbonne Paris Cité, France; 3 Obstetrics and Gynecology Unit, Maternité Port-Royal, APHP, Paris Descartes University, Paris, France; 4 PremUP foundation, Paris, France; 5 DHU Risk and Pregnancy Paris Descartes University, Sorbonne Paris Cité, France; 6 INRA, UMR 1198 Biologie du Développement et Reproduction, F-78350 Jouy en Josas, France

## Abstract

Being able to map accurately placental blood flow in clinics could have major implications in the diagnosis and follow-up of pregnancy complications such as intrauterine growth restriction (IUGR). Moreover, the impact of such an imaging modality for a better diagnosis of placental dysfunction would require to solve the unsolved problem of discriminating the strongly intricated maternal and fetal vascular networks. However, no current imaging modality allows both to achieve sufficient sensitivity and selectivity to tell these entangled flows apart. Although ultrasound imaging would be the clinical modality of choice for such a problem, conventional Doppler echography both lacks of sensibility to detect and map the placenta microvascularization and a concept to discriminate both entangled flows. In this work, we propose to use an ultrafast Doppler imaging approach both to map with an enhanced sensitivity the small vessels of the placenta (~100 μm) and to assess the variation of the Doppler frequency simultaneously in all pixels of the image within a cardiac cycle. This approach is evaluated *in vivo* in the placenta of pregnant rabbits: By studying the local flow pulsatility pixel per pixel, it becomes possible to separate maternal and fetal blood in 2D from their pulsatile behavior. Significance Statement: The *in vivo* ability to image and discriminate maternal and fetal blood flow within the placenta is an unsolved problem which could improve the diagnosis of pregnancy complications such as intrauterine growth restriction or preeclampsia. To date, no imaging modality has both sufficient sensitivity and selectivity to discriminate these intimately entangled flows. We demonstrate that Ultrafast Doppler ultrasound method with a frame rate 100x faster than conventional imaging solves this issue. It permits the mapping of small vessels of the placenta (~100 μm) in 2D with an enhanced sensitivity. By assessing pixel-per-pixel pulsatility within single cardiac cycles, it achieves maternal and fetal blood flow discrimination.

Placental dysfunction is known to be a major cause of pregnancy complications. Inadequate remodeling of the spiral arteries resulting in decreased maternal blood flow to the placenta has been implicated in the pathophysiology of preeclampsia^1^ and IUGR^2^. Maternal utero-placental hemodynamics is currently assessed mainly by means of uterine artery pulsed Doppler, but this imaging modality has limited predictive value for preeclampsia and IUGR^3^. Another approach consists in quantifying the vascularization directly in the placenta or the placental bed using a combined method of three-dimensional (3D) imaging and power Doppler ultrasonography^4^. First clinical studies suggest that the 3D power Doppler indices of the uteroplacental circulation could be helpful to improve the prediction of preeclampsia and IUGR^5^,^6^. However 3D power Doppler angiography of the placenta remains limited to large vessels and does not discriminate the fetal circulation from the maternal circulation. This represents a major limitation because in hemochorial placentation (humans, rabbits, mice) maternal and fetal circulations are strongly entangled in the exchange area and the fetal circulation may easily parasitize the assessment of the maternal placental perfusion.

Recently, the introduction of ultrafast scanners based on holographic imaging using unfocused ultrasonic waves has offered new insight in medical ultrasonic imaging^7^. By increasing the ultrasound frame rate by a factor 100 compared to conventional ultrasound imaging, ultrafast imaging permits to acquire hundreds of temporal samples per second over a large field of view compared to the single spatial location of conventional PW Doppler. Ultrafast acquisition has a particular interest for blood flow imaging and quantification; it offers the possibility to analyze the flow with a high spatio-temporal resolution simultaneously at several locations thus providing new information about the flow behavior.

In this article, the experiments are carried out on pregnant rabbits (n = 4). First, both imaging and quantification capacity of UFD are used to map the placental blood flow. Then, using the same acquisition we demonstrate that the pulsatility of micro vessels (~100 μm) can be evaluated pixel per pixel in the placenta. This is a key advantage as maternal and fetal flows exhibit strongly different pulsatile behaviors. In a second section, an algorithm based on the evaluation of the pulsatility is developed to discriminate maternal and placental blood flow using an automatic thresholding. Finally, the validity of this algorithm is demonstrated by comparison with a real interruption of maternal blood supply during a supra-renal aortic clamp. The side-by-side comparison of the UFD acquisitions performed before and during the supra-renal aortic clamp configuration where only the fetal blood remains permits to evaluate the performance of the separation algorithm on the unclamped acquisition.

## Material and Methods

**Experimental set-up**    *Animals*.    Animal experiments were performed in accordance with the International Guiding Principles for Biomedical Research involving Animals as promulgated by the Society for the Study of Reproduction^8^ and with the European Convention on Animal Experimentation. Researchers involved with direct handling and surgery of the animals possessed an animal experimentation license provided by the French veterinary services. All experimental protocols were approved by National Ethics Committee of Reflection on Animal Experimentation (No12/099).

The *in vivo* experiments were performed on *N* = *4* New Zealand white pregnant rabbits. On day 28 of gestation, i.e., 3 days before the end of gestation, general anesthesia was performed. Ketamine (Imalgène, Merial^®^, France) 35 mg/kg was given intramuscularly for anesthetic induction. Inhaled anesthesia was given via facemask until the rabbit was anesthetized sufficiently, indicated by lack of muscle tone. The endotracheal intubation was performed in direct visualization with a laryngoscope.

The anesthesia was maintained with a mixture of 1–5% isoflurane and 1–1.5 L/min oxygen. Maternal blood pressure was continually monitored with a femoral catheter (BIOPAC Systems, Acknowledge Software, Goleta^®^, USA). The inhaled concentration of isoflurane was continuously adjusted to maintain a mean arterial pressure above 50 mmHg. During the procedure, the rabbit's heart rate and peripheral O_2_ saturation were measured with a pulse oxymeter (BIOPAC Systems, Acknowledge Software, Goleta^®^, USA), and recorded. An abdominal midline laparotomy was performed and both uterine horns were exteriorized. A heating pad was used in order to prevent hypothermia.

**Figure 1:**
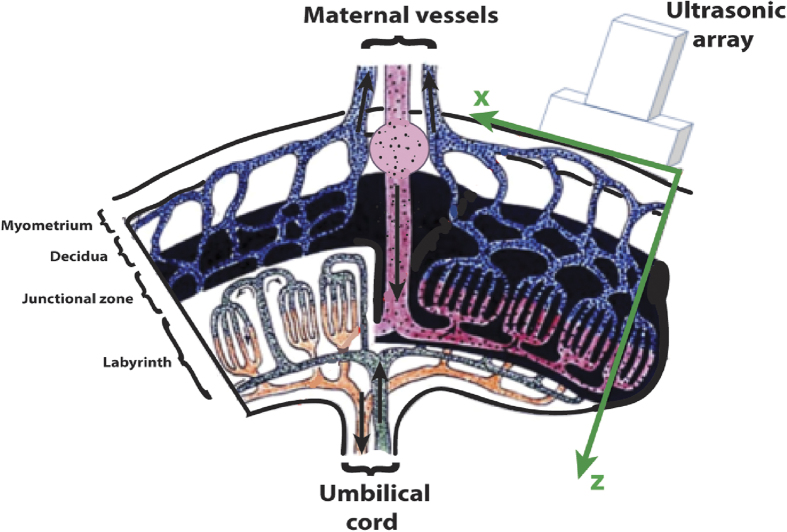
Placement of the ultrasound probe on the rabbit placenta. Modified with permission from Fischer, B. et al. (2012). Rabbit as a reproductive model for human health. Reproduction 144, 1-10.

*Placement of the probe.*    Before each UFD acquisitions, the fetal heart rate (>100 bpm) was systematically checked using the standard PW Doppler of the ultrasound scanner (Aixplorer v6, SuperSonic Imagine, Aix en Provence, France). The 15 MHz linear probe (128 elements, 60% bandwidth) was placed directly in contact with the smaller curvature of the pregnant horns opposite the placental insertion (see Fig. 1). The probe was held stationary with an articulated arm.

**Figure 2:**
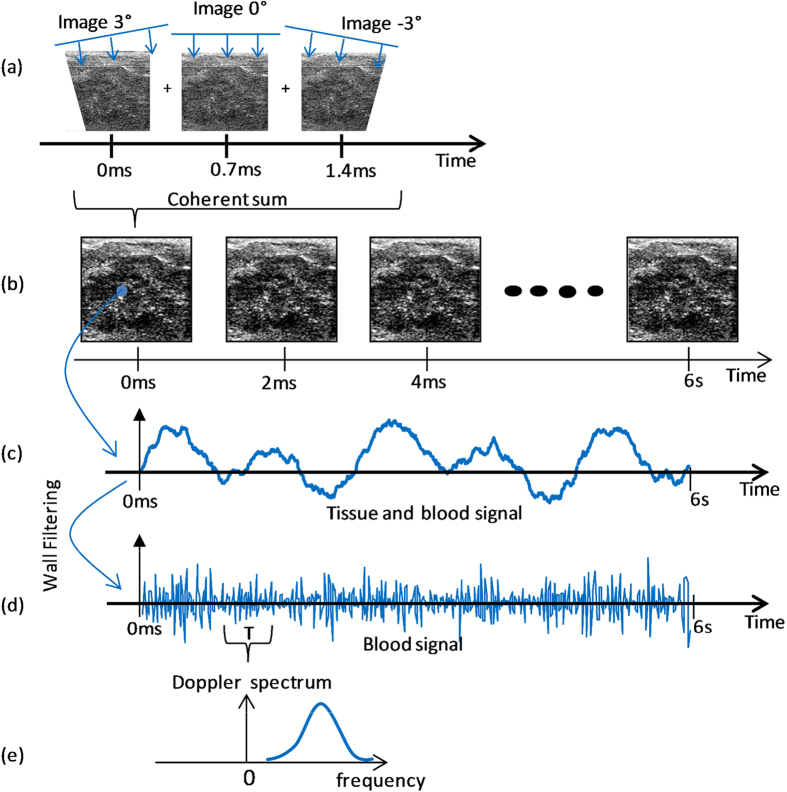
Ultrafast Doppler Sequence. (**a**) Each ultrafast ultrasound image is composed of three tilted plane wave images. (**b**) The placenta is insonified with ultrafast imaging during 6 s. (**c**) From each spatial pixel of the 2D field of view, we can extract a 3000 points temporal signal with tissue and blood signal. (**d**) After wall filtering the blood signal can be extracted. (**e**) At each time T and for each spatial pixel, UFD acquisition permits to compute the PW Doppler spectrum.

**Ultrasound Sequence.**    *Ultrafast Doppler sequence.*    The concept of ultrafast Doppler relies on compounded plane wave transmissions^9^,^10^. The placenta was insonified by an ultrasound plane wave. Then the backscattered echoes were recorded and beamformed to produce an echographic image (see Fig. 2a). The frame rate of this technique can reach more than 10 kHz. However, only a 500 Hz frame rate was needed to sample the blood flow of the placenta. So, in order to increase the signal to noise ratio of each echographic image done at 500 Hz, we managed to compound the echographic images by transmitting several tilted plane waves and summed their backscattered echoes. This compounded sequence permitted to obtain enhanced echographic images leading to a much more sensitive Doppler measurement^11^,^12^. In this article, the UFD ultrasound sequence consisted in transmitting three different tilted plane waves (−3°, 0° and 3°) with a pulse length of 2 cycles at 1500 Hz, then sum coherently their backscattered echoes to produce enhanced echographic images at a frame rate of 500 Hz (see Fig. 2b).

To evaluate accurately blood flow with a high spatial resolution, the ultrasound sequence had to last several fetal heart cycles (~10 heart cycles) so the placenta is insonified during 6 s. At the end of this 6 s acquisition, we obtain 3000 images at 500 Hz (see Fig. 2b).

*Conventional Doppler sequence.*    In order to compare image quality between UFD and conventional Doppler ultrasound, we performed a conventional acquisition of Doppler map. Conventional ultrasound Doppler relies on line per line ultrasound scanning of the medium. Each line among the 128 different lines of the medium is successively sampled by a focused wave with a pulse length of 2 cycles 15 times with a 500 Hz frame rate emitted with the full aperture of the probe. The ultrasound acquisition of one conventional Doppler image is composed of 1920 ultrasound scans and lasted 3.84 s.

**Figure 3:**
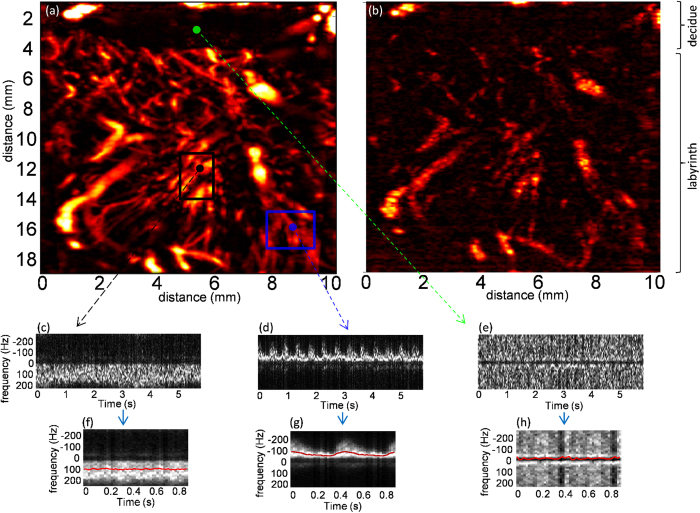
Ultrafast imaging of the placenta. (**a**,**b**) Respectively UDF and conventional Power Doppler image of the placenta containing maternal and fetal blood. (**c**–**e**) UFD PW Doppler of respectively a non pulsatile vessel, a pulsatile vessel and noise within one spatial pixel. (**f**–**h**) Averaged UFD PW Doppler of respectively a non pulsatile vessel, a pulsatile vessel and noise within one spatial pixel.

**Data processing.**    *Wall and tissue motion filtering and Power Doppler.* UFD sequence data. For each spatial pixel of the ultrafast Doppler sequence, we get a 3000 points time signal which contains low frequency tissue signal and high frequency blood signal (Fig. 2c). Using a wall filter (50 Hz high pass Butterworth), the tissue signal has been withdrawn to obtain the blood signal (Fig. 2d). By evaluating the intensity of time signal of each spatial pixel, we obtain a power Doppler image. Figure 3a shows a power Doppler of the placental blood flow containing maternal and fetal blood obtained using 1920 plane wave insonifications of the medium.

Conventional Doppler sequence data. For each spatial pixel of the conventional Doppler sequence, we get a 15 points time signal. Using a wall filter (50 Hz high pass fir filter), the tissue signal has been withdrawn to obtain the blood signal. By evaluating the intensity of time signal of each spatial pixel, we obtain a power Doppler image. Figure 3b shows a power Doppler of the placental blood flow containing maternal and fetal blood obtained using the 1920 focused insonification of the medium.

*The maternal/fetal discrimination algorithm.*    Basic principle. In the rabbit placenta, the maternal blood flows are not pulsatile because the coiled arteries of the endometrium give rise to large arterial sinuses which damp the maternal pulsed pressure^13^. We assume that the maternal flow is not pulsatile whereas the fetus one is. In that case, we can study the time variation of the flow speed at each location of the 2D^13^ image. If the flow speed varies in time (i.e. it is pulsatile), the time variance of the speed is high whereas its time variance is low if the flow speed does not vary in time (i.e. for a non pulsatile flow). In Doppler Ultrasound, the flow speed is proportional to the central frequency (also called Doppler frequency) of the flow Doppler spectrum. For this reason, the discrimination algorithm will be based on an analysis of the time variance of the central frequency (TVCF) of the local flow Doppler spectrum simultaneously in all pixels of the imaged area.

This algorithm can be decomposed in four successive steps.

Step 1: Computing PW Doppler. The first step consists in computing the PW Doppler of each spatial pixel of the ultrasound image. UFD permits to assess the whole ultrasonic Doppler information within one acquisition, giving access both to the Power Doppler image and a local PW Doppler quantification from the signal of each spatial pixel. Let 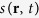 be the complex time signal of a spatial pixel at location 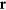 after in phase quadrature demodulation (or Hilbert transform of the raw data). In this article, we will use the normalized time dependent PW Doppler spectrum 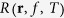 defined as:


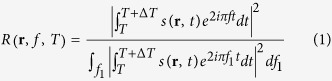


Where T is the time at which the Doppler spectrum is computed using a time window 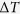 and *f* is the frequency.

Figure 3c,d shows an example of PW Doppler spectrum extracted from one spatial pixel of the ultrafast acquisition (see the point at the beginning of the different arrows on Fig. 3a). As these Doppler spectra are computed using the time signal of one spatial pixel corresponding to a sample volume of 100 μm × 100 μm × 400 μm (lateral distance x depth x elevation distance) to keep a high spatial resolution, they are mainly disturbed by random fluctuation on their amplitude generated by speckle noise. To evaluate the central frequency of this PW Doppler accurately, the acquisition time has to last several cardiac cycles in order to acquire different statistical realizations of this speckle noise. For each spatial pixel, the PW Doppler spectrum of every cardiac cycle can be resynchronized and averaged in order to reduce the random fluctuations on the PW Doppler spectrum. To initiate this process, we first used the information within the ultrasonic data to find the fetal heart cycle as only this fetal flow is pulsatile.

Step II : Finding the fetal heart cycle duration to improve the PW Doppler. The fetal heart cycle can be extracted directly from the ultrafast acquisition. For each spatial pixel at location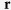, the absolute time signals are auto correlated and the time of maximum correlation 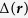 (taken between 0.4 s and 0.8 s corresponding to a heart rate of 150 and 75 bpm respectively) is measured.


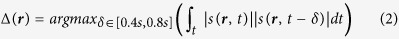


A histogram of 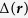 is computed and its maximum 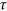 represents the fetal heart cycle duration. The averaged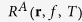 PW Doppler corresponding to the average of the resynchronized cardiac cycles can be written as:


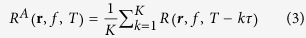


Where K is the number of fetal heart cycles acquired. As seen on Fig. 3f,g, this Doppler spectrum is less disturbed by random fluctuations so the central frequency can be evaluated for each spatial pixel.

Step III: Evaluating the time variance of the central frequency. The time variation of its central frequency 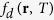 can be computed using the formula of the first order moment of the averaged PW Doppler spectrum:


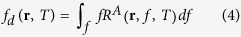


The red curve on Fig. 3f,g shows the evaluation of the fluctuations of the central frequency on the two PW Doppler spectra.

The time variance of the central frequency (TVCF) can then be evaluated for each spatial pixel:


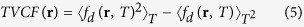


In supplementary methods, equation (39) shows that the TVCF can be written as:


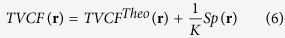


where 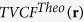 is the theoretical time variance central frequency, without the presence of speckle noise and 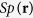 is the influence of the speckle noise. As stated in the section Basic principle, we would like to get a measure of the TVCF the closest possible to the theoretical TVCF so we can detect smaller variations of the blood flow. To that end, we have to reduce the amplitude of the speckle noise in the measured TVCF i.e. the term 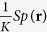 in equation (6). One way to reduce the speckle noise in ultrasound is to compute incoherent averaging of several spatial pixels however this solution will lead to a decrease of spatial resolution. Giving a closer look at the term 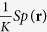 in equation (6), it shows that averaging the PW Doppler spectra on K heart cycles will decrease the speckle noise term. Averaging on several cardiac cycles allows us to detect smaller variations of the blood flow without decreasing the spatial resolution. That is the reason why the ultrasound acquisition lasted several heart cycles.

Step IV: Automatic thresholding on the TVCF. The main question of this section is: How can we threshold automatically the TVCF data in order to separate pulsatile and non pulsatile pixels? As the influence of the speckle noise has been reduced by averaging several Doppler spectra, we hypothesize that the TVCF of pulsatile flows is superior to the TVCF of non pulsatile flow which are only generated by speckle noise. The aim is now to find the maximum TVCF for a non pulsatile flow.

For non pulsatile flows, 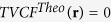, so the 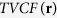 is entirely due to the speckle noise variation and can be written using equation (41) in supplementary methods as:


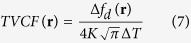


where 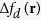 is the bandwidth of the PW Doppler spectrum of the non pulsatile flow. 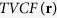 is maximal if 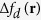 is maximal. That is the case with spatial pixels containing no blood signal. These pixels contain only noise mostly generated by the electronic of the echographic system: If we consider that the noise is white 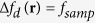 with 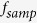 being the sampling frequency (500 Hz in our case). We also hypothesize that the amplitude of the electrical noise is negligible compared to the amplitude of the speckle noise within the spatial pixels containing blood signal. Figure 3e shows the PW Doppler extracted from a spatial pixel containing no blood signal, i.e. only electrical noise and Fig. 3h shows its averaged PW Doppler.

To summarize, we can classify the different measured TVCF as:


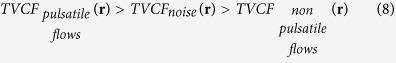


**Figure 4:**
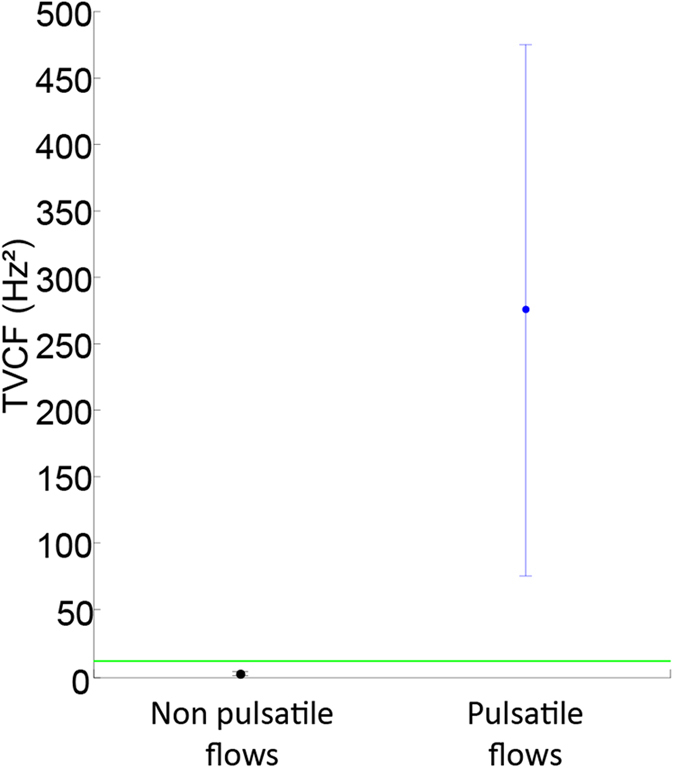
TVCF of pulsatile and non pulsatile flows. The green dotted line represents the TVCF of the noise. The black point (+/− standard deviation) represents the distribution of the TVCF of non pulsatile flows. The blue point (+/− standard deviation) represents the distribution of the TVCF of pulsatile flows.

To avoid the theoretical approximation done to calculate the pre factor 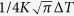 of the TVCF of the noise^14^, we can directly retrieve the TVCF of the noise from the data. We compute the TVCF for every location 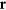 in the image, as most of spatial pixels signals correspond to noise, the median of all TVCF will give the value of the TVCF of the noise. This displayed on Fig. 4 as a green dotted line.

Figure 4 also shows two examples of TVCF distribution computed on two different regions. In back (see Fig. 4), a region containing only non pulsatile flows (see the area limited by a black rectangle on Fig. 3a), we see that the mean value of this TVCF distribution (+/– its standard deviation) is below the threshold value i.e. the TVCF of the noise (green dotted line). In blue (see Fig. 4), a region containing only pulsatile flows (see the area limited by a blue rectangle on Fig. 3a), we notice that the mean value of this TVCF distribution (+/– its standard deviation) is over the threshold value i.e. the TVCF of the noise (green dotted line).

The distributions of TVCF of pulsatile and non-pulsatile flows were found significantly different using a Wilcoxon rank sum test (p < 0.001).

**Experimental Results**

**Mapping blood flow into the placenta.**    Figure 3a,b compares the power Doppler image of the placenta obtained respectively with an UFD sequence and a conventional ultrasound sequence using the same amount of energy transmitted in the medium (1920 insonications of the medium). Both power Doppler images can be decomposed into the decidue (first two mm deep) containing maternal blood and the labyrinth containing maternal and fetal blood flows. On both power Doppler images, we can image the large vessels into in the decidue however due to a higher sensitivity of UFD compared to conventional Doppler we clearly notice more small vessels in the labyrinth of the placenta on the UFD power Doppler image. We can notice the resolution of very small vessels (~100 μm) of UFD. UFD provides a higher sensitive mapping of the blood flow of the two parts of the placenta (decidue and labyrinth) than conventional Doppler.

**Figure 5:**
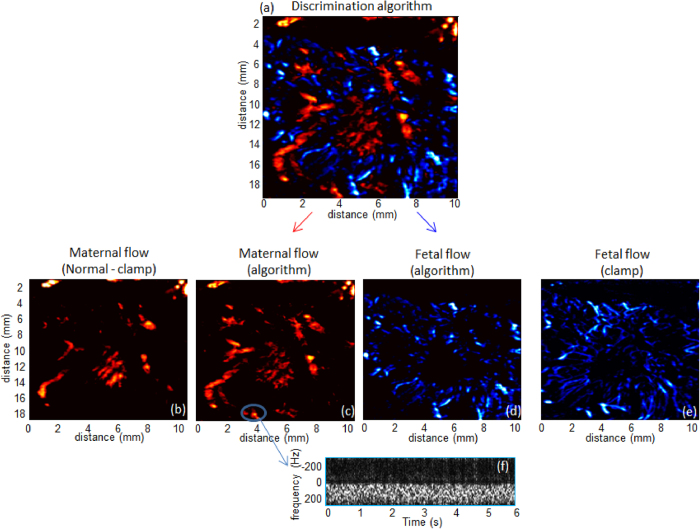
Discrimination of maternal and fetal flows. (**a**) Results of the discrimination of fetal blood (blue) and maternal blood (red). (**b**) Maternal blood computed from the difference between the UFD image before and after the maternal aortic clamping. (**c**) Maternal blood computed from the pulsatility discrimination algorithm. (**d**) Fetal blood computed from the pulsatility discrimination algorithm. (**e**) UFD Power Doppler image of fetal blood acquired after the aortic clamping. (**f**) UFD PW Doppler extracted from the vessel circled with a blue line on (**c**).

**Result of the discrimination algorithm.**    Figure 5a shows the result of the discrimination algorithm between maternal and fetal flow based on the local flow pulsatility. Pulsatile flows (assumed to be fetal) are displayed in blue whereas non pulsatile flows (assumed to be maternal) are displayed in red. In the labyrinth we can notice that the part of the blood flow found as fetal is non negligible compared to the part of the blood flow found as maternal.

**Comparison with the maternal aortic clamp experiment.**    Figure 5b shows the difference between the UDF Power Doppler image just before and after the aortic clamp. By this way, it displays only the maternal blood flows retrieve by the aortic clamp experiment. Figure 5e shows the UFD Power Doppler image after the maternal aortic clamp, this image displays the fetal blood flows, and for sake of clarity the power Doppler image is showed using a blue color map.

We divided the image of the discrimination algorithm into two separated images, one displaying the non-pulsatile flows (Fig. 5c), and one displaying the pulsatile flows (Fig. 5d).

There is a systematic matching between the maternal blood flows deduced after the aortic clamping (Fig. 5b) and the non-pulsatile blood flows found by the discrimination algorithm (Fig. 5c). In the same way, there is a systematic matching between the fetal blood flows observed after the aortic clamping (Fig. 5e) and the pulsatile blood flows found by the discrimination algorithm (Fig. 5d). However some small areas as the one circled with a blue line on Fig. 5c, classified as non-pulsatile blood flow by the discrimination algorithm does not show on the maternal blood flow found by the aortic clamping experiment (Fig. 5b). The reason of this difference is simple to understand if we compute the UFD PW Doppler of these vessels. Indeed, we clearly notice that the flow is non-pulsatile in these regions (Fig. 5f).

## Discussion

### Discrimination of maternal and fetal blood flows in the rabbit placenta

In this study UFD allowed us to map maternal and fetal blood flow into the placenta with a higher sensitivity than conventional Doppler imaging. As previously demonstrated^12^, UFD can be 5 to 30 times more sensitive to flows than conventional Doppler techniques. UFD can detect multidirectional and slow maternal blood flows in the labyrinth area of the rabbit placenta, this technology could be more efficient than the conventional Doppler for the exploration of the maternal circulation in human placenta.

UFD also managed to discriminate maternal and fetal blood flow by taking benefit of its high spatio-temporal resolution. The proposed signal processing was based on the evaluation of the pulsatility of the micro vascularization assuming that the fetal blood flows in the placenta are pulsatile and that the placental maternal blood flows are continuous. We built an algorithm based on the detection of pulsatility in order to discriminate the maternal circulation and the fetal circulation with an automatic thresholding. This algorithm requires only a few cardiac cycles acquisitions (~6 s) and calculations can be performed in a very short time (<10 s on a 3 GHz, 6 cores processor) in post-processing just after the acquisition.

We can also notice that the pulse repetition frequency of UFD of the ultrasound sequence programmed in this article (1.5 kHz i.e. 3 plane waves with different angles at 500 Hz) is not maximal and can reach more than 10 kHz^11^. More plane waves with different angles can be added to produce a high quality echographic image at 500 Hz to increase the sensitivity of UFD^12^. This limitation is due to the software used to program the echographic system and will be soon overcome.

In this study we developed and validated in the rabbit placenta, an algorithm for discrimination of maternal and fetal circulation based on the difference of pulsatility of arterial blood flows. In Fig. 5a, the blood flows detected at a distance of two millimeters of the probe are maternal. Since the tissues located within two millimeters of the probe correspond to the maternal decidue, these areas are therefore vascularized only by the mother. The experiences of maternal aortic clamping have demonstrated the effectiveness of the discrimination algorithm in the areas of the placenta where the maternal and fetal circulations are close. The pulsatility algorithm is efficient and most vessels were classified on the right category.

Finally, some placental areas detected as maternal blood flows by the algorithm, are still detected after the maternal aortic clamp (Fig. 5f). The PW Doppler of these areas is non pulsatile so this artifact is not due to the discrimination algorithm. This artifact is generated by the blood flows in the large fetal veins of the chorionic plate which are not pulsatile; this was verified by a standard PW Doppler. This explains the mismatch between the maps obtained by the algorithm and the maps obtained after aortic clamping. This is a limitation of the algorithm developed in this study that it does not allow to discriminate the maternal continuous blood flows with the fetal venous continuous blood flows in the large chorionic veins. The signal processing algorithm still needs to be improved to detect exclusively the human maternal intraplacental blood flows but this first step is extremely promising (see Fig. 5).

**Potential clinical relevance of UFD in the human placenta.**    In uteroplacental hypoxia, maternal oxygenation is normal, but because of impaired uteroplacental circulation the placenta and fetus are both hypoxic^15^. In this situation, peripheral placental villi similarly show the formation of richly branching capillary networks and fetal blood flow impedance is normal or even reduced. In postplacental hypoxia, the fetus is hypoxic whereas the mother is normoxic and the placenta may show even higher PO_2_ levels than normal, a situation described as placental hyperoxia. In this situation, the terminal villus capillaries are poorly developed, capillary branching is virtually absent, and the resulting fetoplacental flow impedance is considerably increased. The UFD technology, discriminating fetal and maternal placental circulation would detect and differentiate these two hemodynamic situations. Clinical studies with the UFD could confirm the hypothesis of the uteroplacental and postplacental hypoxia and understand why preeclampsia and IUGR are two inconsistently associated pathologies.

As UFD has higher sensitivity than conventional Doppler, we could quantify more accurately vascular indices in the human placenta. In fact, vascularization index (VI) which reflects the relative proportion of color voxels to gray voxels or the flow index (FI) which represents the mean power Doppler signal intensity value have shown a significant correlation with the maternal flow rate^16^. The values of these indices could also be improved by discriminating maternal and fetal flows. As shown by Demene *et al.*^17^ in neonate brain, vascular resistivity index of the spiral jet flows entering the placenta could be evaluate in 2D as these flows are known to be pulsatile^18^. This could also help the diagnosis and follow-up of pregnancy complications.

This differential approach based on pulsatility could also be applied in the placenta of the monochorionic twin pregnancies^19^. The two fetal heart rates are asynchronous and the algorithm could discriminate automatically the two frequencies. After an acquisition of a 3D placental volume, it could be therefore possible to estimate the placental fraction vascularized by each twin. This fraction could be evaluated as an indicator of adverse perinatal outcome.

In the hemochorial placentas, over 50% of the placental volume consists of maternal blood. Maternal blood circulates in the intervillous space (human) or in the labyrinth (rabbit) according to physical laws probably closer to the percolation in a porous medium than to the perfusion in a capillary network^20^. This explains why the velocities of maternal blood in the intervillous space (IVS) and in the labyrinth are very low despite a high total flow rate. In the IVS the average pore diameter is estimated to be 100–150 microns and the average speed of the maternal blood was calculated to 0.6 mm/s^21^. These circulatory modes are undetectable by conventional Doppler techniques. Currently no ultrasound technique allows to detect the maternal hemodynamics disruptions (placental infarctions, intervillous thrombosis, villous fibrinoïd necrosis, placental abruption) *in vivo* directly inside the placenta. Detection and quantification of maternal placental vasculature remains a major challenge for clinicians, and faces the difficulty of detecting very slow and multidirectional blood flows in 3D. Maternal utero-placental hemodynamics are assessed indirectly by means of uterine artery pulsed Doppler, but this imaging modality has limited predictive value for preeclampsia and IUGR and does not inform the clinicians on intraplacental anomalies. The UFD technology can detect very slow and multidirectional flows. Applied to the human placenta this technology could explore the intraplacental flows but the discrimination of maternal and fetal circulations will be an essential step. Automatic discrimination algorithms could be developed by exploiting the differences of maternal and fetal circulatory modes like average velocities of the flows, directionality of the flows or pulsatility of the flows.

**Potential clinical limitations of UFD in the human placenta**.    In this article, we have developed an automatic discrimination algorithm based on the differences of pulsatility. This approach will be insufficient in the human placenta. The pulsatility of the intraplacental maternal flows is quickly damped in the IVS and an algorithm only based on pulsatility difference will not allow us to distinguish the maternal circulation of fetal venous circulation. Discrimination by the pulsatility will initially isolate fetal arterial Doppler signal. Other parameters will be necessary to assess to differentiate the maternal circulation fetal venous return. A new discrimination algorithm would also have to take in account the maternal pulsatility of the spiral jet flows entering the placenta.

We also noticed that during the 6 seconds UFD acquisition sequence the fetus motion can occur. However, most of the placental motion is due to the pregnant woman respiration. We can easily ask the patient to block her respiration during a few second. Yet, this acquisition sequence still needs to be improved and shortened to be able to acquire 3D placental volumes using a wobbling probe as developed by Raine-Fenning^22^.

## Conclusion

The ultrafast Doppler (UFD) technology provides a two dimensional and high sensitivity map of the placental vascularization without requiring any contrast agent injection. The UFD acquisitions offers key features beyond the state of the art in placental imaging. Firstly, it computes a PW Doppler in each spatial pixel, and secondly it evaluates the pulsatility accurately and simultaneously in all pixels or voxels of the image. An algorithm based on the analysis of the time variance of the central Doppler frequency leads to the discrimination of maternal and fetal blood in 2D from their pulsatile behavior. Finally, the advent of GPU (Graphics Processing units) technology and the Moore's law for computational power evolution makes it possible to envision future applications of this approach in 3D in a very near future.

## Additional Information

**How to cite this article**: Osmanski, B.-F. *et al.* Discriminative imaging of maternal and fetal blood flow within the placenta using ultrafast ultrasound. *Sci. Rep.* **5**, 13394; doi: 10.1038/srep13394 (2015).

## Supplementary Material

Supplementary Methods
