# Supplementary material for: Discriminative imaging of maternal and fetal blood flow within the placenta using ultrafast ultrasound
Source: Sci Rep. 2015 Sep 16;5:13394. doi: 10.1038/srep13394 (PMC4570988; doi:10.1038/srep13394)
Supplement: Supplementary Methods [file srep13394-s1.pdf]

Discriminative imaging of Maternal and fetal blood flow within the placenta using Ultrafast Ultrasound

Bruno-Felix Osmanski[1], Edouard Lecarpentier[2-3-4-5], Gabriel Montaldo[1], Vassilis Tsatsaris[3-4-5], Pascale Chavatte-Palmer[4-6-7], Mickael Tanter[1]

[1] Institut Langevin, ESPCI ParisTech, PSL University, CNRS UMR7587, INSERM U979, Université Paris VII, France.
[2] UMR-S 1139 INSERM, Paris Descartes University , Sorbonne Paris Cité, France
[3] Obstetrics and Gynecology Unit, Maternité Port-Royal, APHP, Paris Descartes University, Paris, France
[4] PremUP foundation, Paris, France
[5] DHU Risk and Pregnancy Paris Descartes University , Sorbonne Paris Cité, France
[6] INRA, UMR 1198 Biologie du Développement et Reproduction, F-78350 Jouy en Josas, France
[7] CRII and UCEA, INRA, Jouy-en-Josas, France

Supplementary Methods

Theoretical analysis of the pulsatility discrimination algorithm

This supplementary section details the theoretical basis of the blood flow classification as pulsatile or non pulsatile using the TVCF computed from the improved PW Doppler. The first part is dedicated to the statistics of the speckle noise on Doppler spectra. In a second part, the theoretical TVCF without speckle noise will be studied then the measured TVCF will be expressed. In the last part, the measured TVCF will be studied in the case of a non pulsatile flow to set an automatic thresholding between the pulsatile and non pulsatile flows.

Statistics of the speckle noise

The mean time value

The aim of this paragraph is to calculate the statistical mean value of the speckle noise on the normalized Doppler spectrum.
We start by introducing the normalized (using the energy) PW Doppler:

$$R(\mathbf{r},f,T) = \frac{\left|\int_T^{T+\Delta T} s(\mathbf{r},t)e^{2i\pi f t}dt\right|^2}{\int_{f_1}\left|\int_T^{T+\Delta T} s(\mathbf{r},t)e^{2i\pi f_1 t}dt\right|^2 df_1} \tag{9}$$

so

$$\int_f R(\mathbf{r},f,T)df = 1 \tag{10}$$

$R(\mathbf{r},f,T)$ can be written as:

$$R(\mathbf{r},f,T) = H(\mathbf{r},f,T)\chi(\mathbf{r},f,T) \tag{11}$$

with $H(\mathbf{r},f,T)$ being the normalized PW Doppler spectrum without speckle noise and $\chi(\mathbf{r},f,T)$ representing the speckle noise perturbation acting as a multiplicative noise. As a consequence of equation (10) and (11):

$$\int_f H(\mathbf{r},f,T)\chi(\mathbf{r},f,T)df = 1 \quad (12)$$

By taking the average value of the time dimension ($\langle\ \rangle_T$) previous equation gives:

$$\langle \int_f H(\mathbf{r},f,T)\chi(\mathbf{r},f,T)df \rangle_T = 1 \quad (13)$$

As the theoretical spectrum $H(\mathbf{r},f,T)$ and the speckle $\chi(\mathbf{r},f,T)$ are independent:

$$\int_f \langle H(\mathbf{r},f,T)\rangle_T \langle \chi(\mathbf{r},f,T)\rangle_T df = 1 \quad (14)$$

Due to the fact that $H(\mathbf{r},f,T)$ is normalized ($\int_f \langle H(\mathbf{r},f,T)\rangle_T df = 1$) and that the statistics of speckle noise do not depend on the time or the frequency, we deduce that:

$$\langle \chi(\mathbf{r},f,T)\rangle_T = 1 \quad (15)$$

To simplify the calculus, we can introduce φ:

$$\chi(\mathbf{r},f,T) = 1 + \varphi(\mathbf{r},f,T) \quad (16)$$

So that:

$$\langle \varphi(\mathbf{r},f,T)\rangle_T = 0 \quad (17)$$

The correlation values

As the speckle noise is assumed to be completely decorrelated at two different times, it is also decorrelated at two different frequencies, so we can write:

$$\langle \varphi(\mathbf{r},f_1,T_1)\varphi(\mathbf{r},f_2,T_2)\rangle_T = \sigma(\mathbf{r})\delta(f_1-f_2)\delta(T_1-T_2) \quad (18)$$

with $\sigma(\mathbf{r})$ representing the variance of the speckle noise and can be associated with the reflectivity of the medium.

TVCF without speckle noise

The aim of this part is to evaluate the theoretical TVCF without the presence of speckle noise:

The central frequency can be evaluated using the first order momentum of theoretical normalized spectrum $H(\mathbf{r},f,T)$:

$$f_d^{Theo}(\mathbf{r},T) = \int_f fH(\mathbf{r},f,T)df \quad (19)$$

TVCF (time variance of the central frequency) can be written as:

$$TVCF^{Theo}(\mathbf{r}) = \langle f_d^{Theo}(\mathbf{r},T)^2\rangle_T - \langle f_d^{Theo}(\mathbf{r},T)\rangle_T{}^2 \quad (20)$$

So:

$$TVCF^{Theo}(\mathbf{r}) = \langle \left(\int_f fH(\mathbf{r},f,T)df\right)^2\rangle_T - \langle \int_f fH(\mathbf{r},f,T)df\rangle_T{}^2 \quad (21)$$

which reduces into:

$$TVCF^{Theo}(\mathbf{r}) = \iint_{f_1,f_2} f_1 f_2 \langle H(\mathbf{r},f_1,T) H(\mathbf{r},f_2,T)\rangle_T df_1 df_2 - \left(\int_f \ f\langle H(\mathbf{r},f,T)\rangle_T\, df\right)^2 \quad (22)$$

TVCF with speckle noise

In this paragraph, we aim at expressing the measured TVCF.

Evaluating the central frequency versus time

We start by introducing the averaged PW Doppler spectrum:

$$R^A(\mathbf{r},f,T) = \frac{1}{K}\sum_{k=1}^{K} R(\boldsymbol{r},f,T-k\tau) \quad (23)$$

Using equation (11):

$$R^A(\mathbf{r},f,T) = \frac{1}{K}\sum_{k=1}^{K} H(\mathbf{r},f,T-k\tau)\chi(\mathbf{r},f,T-k\tau) \quad (24)$$

where K is number of heart cycles averaged and τ is the heart cycle duration. As $H(\mathbf{r},f,T)$ does not depend on which heart cycle it is evaluated:

$$H(\mathbf{r},f,T-k\tau) = H(\mathbf{r},f,T) \quad (25)$$

so equation (24) reduces into:

$$R^A(\mathbf{r},f,T) = H(\mathbf{r},f,T)\frac{1}{K}\sum_{k=1}^{K} \chi(\mathbf{r},f,T-k\tau) \quad (26)$$

The measured central frequency can be computed using the first order momentum formula:

$$f_d(\mathbf{r},T) = \int_f \ fR^A(\mathbf{r},f,T)df \quad (27)$$

so using equation (26):

$$f_d(r,T) = \int_f \ H(\mathbf{r},f,T)\frac{1}{K}\sum_{k=1}^{K} \chi(\mathbf{r},f,T-k\tau)\, df \quad (28)$$

Evaluating the TVCF with speckle noise

The TVCF represents the time variance of the measured central frequency so it can be expressed as:

$$TVCF(\mathbf{r}) = \langle f_d(\mathbf{r},T)^2\rangle_T - \langle f_d(\mathbf{r},T)\rangle_T{}^2 \quad (29)$$

so we need to compute $\langle f_d(\mathbf{r},T)^2\rangle_T$ and $\langle f_d(\mathbf{r},T)\rangle_T$.

$$\langle f_d(\mathbf{r},T)^2\rangle_T = \langle\left(\int_f \ fH(\mathbf{r},f,T)\frac{1}{K}\sum_{k=1}^{K} \chi(\mathbf{r},f,T-k\tau)\, df\right)^2\rangle \quad (30)$$

so using the independency of χ and H, we can write:

$$\langle f_d(\mathbf{r},T)^2\rangle_T = \frac{1}{K^2}\sum_{k_1}^{K}\sum_{k_2}^{K}\iint_{f_1,f_2} \langle H(\mathbf{r},f_1,T)H(\mathbf{r},f_2,T)\rangle_T \langle\chi(\mathbf{r},f_1,T-k_1\tau)\chi(\mathbf{r},f_2,T-k_2\tau)\rangle_T d\, f_1 df_2 \quad (31)$$

We introduce φ:

So $\langle\chi(\mathbf{r},f_1,T-k_1\tau)\chi(\mathbf{r},f_2,T-k_2\tau)\rangle_T$ in equation (31) can be written as:

$$\langle \chi(\mathbf{r}, f_1, T - k_1\tau)\chi(\mathbf{r}, f_2, T - k_2\tau)\rangle_T$$
$$= 1 + \langle \varphi(\mathbf{r}, f_1, T - k_1\tau)\rangle_T + \langle \varphi(\mathbf{r}, f_2, T - k_2\tau)\rangle_T$$
$$+\langle \varphi(\mathbf{r}, f_1, T - k_1\tau)\varphi(\mathbf{r}, f_2, T - k_2\tau)\rangle_T \quad (32)$$

Using equation (31) and (32) it reduces to:

$$\langle f_d(\mathbf{r}, T)^2\rangle_T = \iint_{f_1, f_2} f_1 f_2 \langle H(\mathbf{r}, f_1, T) H(\mathbf{r}, f_2, T)\rangle_T df_1 df_2 + \frac{\sigma(\mathbf{r})}{K}\int_f f^2 \langle H(\mathbf{r}, f, T)^2\rangle_T df \quad (33)$$

Now, we can analyze $\langle f_d(\mathbf{r}, T)\rangle_T$:

$$\langle f_d(\mathbf{r}, T)\rangle_T = \langle \int_f f H(\mathbf{r}, f, T)\frac{1}{K}\sum_{k=1}^{K} \chi(\mathbf{r}, f, T - k\tau)\, df\rangle_T \quad (34)$$

The previous equation becomes:

$$\langle f_d(\mathbf{r}, T)\rangle_T = \int_f f\langle H(\mathbf{r}, f, T)\rangle_T \frac{1}{K}\sum_{k=1}^{K}\langle \chi(\mathbf{r}, f, T - k\tau)\rangle_T\, df \quad (35)$$

So using equation (15) it reduces to:

$$\langle f_d(\mathbf{r}, T)\rangle_T = \int_f f\langle H(\mathbf{r}, f, T)\rangle_T\, df \quad (36)$$

By combining eq. (15), (22); (33), the measured TVCF can be expressed as:

$$TVCF(\mathbf{r}) = TVCF^{Theo}(\boldsymbol{r}) + \frac{\sigma(\mathbf{r})}{K}\int_f f^2\langle H(\mathbf{r}, f, T)^2\rangle_T df \quad (37)$$

We define the influence of the speckle noise (without averaging on different cardiac cycles) as:

$$Sp(\mathbf{r}) = \sigma(\mathbf{r})\int_f f^2\langle H(\mathbf{r}, f, T)^2\rangle_T df \quad (38)$$

We can conclude that:

$$TVCF(\mathbf{r}) = TVCF^{Theo}(\boldsymbol{r}) + \frac{Sp(\mathbf{r})}{K} \quad (39)$$

<u>TVCF of a non pulsatile flow</u>

To set an automatic thresholding between pulsatile and non pulsatile flows, the TVCF of a non pulsatile flow is studied.
For each location $\boldsymbol{r}$, as the flow is non pulsatile $TVCF^{Theo}(\boldsymbol{r}) = 0$. So the TVCF is only due to the speckle noise. In [14], it is proven that the spatial standard deviation of the central frequency is (equation (8)):

$$\Delta f^{DFSA} = \frac{1}{2\pi^{1/4}}\sqrt{\frac{\Delta f_d f_{samp}}{N}} \quad (40)$$

where Δf_d is the bandwidth of the Doppler spectrum, Δf_{samp} is the sampling frequency and N is the number of points used to compute the Doppler spectrum. In this article, we do not use different spatial realizations for the central frequency, however, we can assume the ergodicity of the speckle noise so the spatial realizations can be transformed into temporal

realizations. In this article, all Doppler spectra are computed using the same time window $\Delta T = N/\Delta f_{samp}$ so the previous formula can be adapted in our case into:

$$TVCF(r) = \frac{\Delta f_d(\mathbf{r})}{4K\sqrt{\pi}\Delta T} \quad (41)$$
